# Silencing of the hTERT Gene by shRNA Inhibits Colon Cancer SW480 Cell Growth *In Vitro* and *In Vivo*

**DOI:** 10.1371/journal.pone.0107019

**Published:** 2014-09-10

**Authors:** Ai-Qun Liu, Lian-Ying Ge, Xiao-Ling Lu, Xiao-Ling Luo, Yan-Ling Cai, Xing-Qing Ye, Fang-Fang Geng

**Affiliations:** 1 Department of Endoscopy, The Affiliated Tumor Hospital of Guangxi Medical University, Nanning, Guangxi, P.R. China; 2 National Center for International Research of Biological Targeting Diagnosis and Therapy, Guangxi Key Laboratory of Biological Targeting Diagnosis and Therapy Research, Guangxi Medical University, Nanning, Guangxi, P.R. China; 3 Science and Technology Agency, Guangxi Medical University, Nanning, Guangxi, P.R. China; 4 Department of Pathology, The Affiliated Tumor Hospital of Guangxi Medical University, Nanning, Guangxi, P.R. China; Wayne State University School of Medicine, United States of America

## Abstract

Human telomerase reverse transcriptase (hTERT) is the key enzyme responsible for synthesizing and maintaining the telomeres on the ends of chromosomes, and it is essential for cell proliferation. This has made hTERT a focus of oncology research and an attractive target for anticancer drug development. In this study, we designed a small interfering RNA (siRNA) targeting the catalytic subunit of hTERT and tested its effects on the growth of telomerase-positive human colon carcinoma SW480 cells in vitro, as well as on the tumorigenicity of these cells in nude mice. Transient and stable transfection of hTERT siRNA into colon cancer SW480 cells suppressed hTERT expression, reduced telomerase activity and inhibited cell growth and proliferation. Knocking down hTERT expression in SW480 tumors xenografted into nude mice significantly slowed tumor growth and promoted tumor cell apoptosis. Our results suggest that hTERT is involved in carcinogenesis of human colon carcinoma, and they highlight the therapeutic potential of a hTERT knock-down approach.

## Introduction

Colorectal carcinoma is the third most common cancer worldwide and the fourth most common cause of death [1]–[2]. In 2008 alone, approximately 1.23 million new cases of colorectal cancer were diagnosed around the world, and 608,000 people died from the disease [3]. The standard treatment for this cancer is surgical, but outcomes are far from satisfactory, with up to 50% of patients suffering recurrence or death within 5 years of surgery [4].

Targeting telomerase in colon carcinoma may provide an effective alternative or complement to surgical treatment. Telomerase, a ribonucleoprotein complex containing an internal RNA template (hTR) and a catalytic protein with telomere-specific reverse transcriptase activity (hTERT), extends telomeres at the end of eukaryotic chromosomes, thus preventing cell senescence and death. Telomerase appears to play a key role in tumor growth and proliferation: expression and activity of the enzyme are abnormally elevated in most cancers [5]–[6], and down-regulating the enzyme inhibits growth and proliferation [7]. While hTR is constitutively present in normal and tumor cells, hTERT is the rate-limiting component of the telomerase complex, and its expression correlates with telomerase activity [8]. In normal somatic tissues, hTERT activity is repressed, but both hTERT expression and telomerase activity are elevated in most human tumors [9]–[10]. Several studies indicate that telomerase may be key to immortalizing cells as a necessary step in oncogenesis [11]–[12], making hTERT a potentially useful clinical biomarker [13] and target for anticancer research [14].

In colorectal cancer, up to 85% of cells contain active telomerase, whereas only about 5% of normal colorectal cells contain active enzyme. Therefore targeting the expression or activity of telomerase may provide a novel therapy for colorectal cancer. Given that no highly selective telomerase inhibitors are available for treating any cancer, we focused on gene therapy approaches. Gene therapy is expected to play a key role in next-generation cancer therapy in conjunction with conventional treatments such as surgery, chemotherapy, and radiotherapy [15]. One gene therapy is RNA interference (RNAi), which can down-regulate (“knock-down”) the expression of specific genes, allowing the functions of the genes to be analyzed or blocked for therapeutic purposes [16]–[18].

In the present study, we designed a novel hTERT small interfering RNA (siRNA) and expressed the corresponding short hairpin RNA (shRNA) in human colorectal cells in vitro and in nude mice. We found that knocking down hTERT expression inhibited human colon carcinoma cell growth, raising the possibility of gene therapy approaches that target hTERT.

## Materials and Methods

### Cell culture

Human colon carcinoma cell lines SW480, DLD-1, and HT29 (Academia Sinica Cell Bank, Shanghai, China) were grown in low-glucose Dulbecco’s modified Eagle medium (SW480) or RPMI-1640 medium (DLD-1 and HT29) (GibcoBRL, Grand Island, NY, USA) supplemented with 10% (v/v) fetal bovine serum, 100 IU/mL penicillin, and 10 mg/mL streptomycin. Cultures were incubated in 5% CO_2_ at 37°C.

### Ethics Statement and Animals

This study was carried out in strict accordance with the Guide for the Care and Use of Laboratory Animals of the U.S. National Institutes of Health, and the study protocol was approved by the Committee on the Ethics of Animal Experiments of Guangxi Medical University. All surgeries was performed under sodium pentobarbital anesthesia, and suffering was minimized as much as possible. Athymic nude mice (BALB/cA nu/nu) aged 4–5 weeks (Guangxi Institute of Materia Medica, Nanning, China) were housed in sterile cages under laminar airflow hoods in a specific pathogen-free room with a 12 h:12 h light-dark schedule. Animals were fed autoclaved chow and water ad libitum.

### RT-PCR to measure hTERT mRNA expression in different cell lines

Total RNA was extracted from SW480, DLD-1 and HT29 cells using Trizol (Bio Basic Inc., Canada) according to the manufacturer’s instructions. First-strand cDNA synthesis was carried out with 2 µg RNA from each cell line and Moloney murine leukemia virus RT (MMLV-RT; MBI Fermentas, Amherst, NY, USA), then amplified in a 50-µl reaction mixture containing 50 mM of each of the four dNTPs, 3 U of Taq DNA polymerase (Promega), and 0.5 mM of primers (Sangon Co., Shanghai, China). The primers 5′-GCTGCTCAGGTCTTTCTTTTATG-3′ and 5′-CGACGTAGTCCATGTTCACAA-3′ were used to amplify a 252-bp region within the hTERT gene. As an internal control, expression of glyceraldehyde phosphate dehydrogenase (GAPDH) was assayed using the primers 5′-CTCAGACACCATGGGGAAGGTGA-3′ and 5′-ATGATCTTGAGGCTGTTGTCATA-3′ to amplify a 450-bp region. Amplification reactions were performed for 30 cycles of 94°C for 30 s, 56°C for 45 s and 72°C for 45 s. PCR products were separated on a 2.0% agarose gel, which was stained with nucleic acid stain I (Roche Diagnostics, Mannheim, Germany), photographed and analyzed semi-quantitatively using GeneTools Analysis Software (Syngene, Cambridge, UK). The cell line showing the highest level of hTERT mRNA was chosen for further study.

### Design of siRNAs targeting hTERT

Three hTERT siRNA sequences were designed as described [19]. The sequences were as follows, with nucleotide start positions based on the NCBI entry for hTERT (accession NM_198253): hTERT-966, 5′-CGGUGUACGCCGAGACCAATT-3′;

hTERT-2862, 5′-GAGCCAGUCUCACCUUCAATT-3′; and hTERT-2985, 5′-CGGUGUGCACCAACAUCUATT-3′. In parallel, we designed an unrelated sequence as a negative control for siRNA specificity: 5′-UUCUCCGAACGUGUCACGUTT-3′. All four sequences were submitted to a BLAST search against the human genome to exclude the possibility of significant homology to other genes. The sequences were chemically synthesized (GenePharma Co., Shanghai, China) and transiently transfected into SW480 cultures that had been cultured overnight to 40–60% confluence in complete medium without antibiotics. Transfections were performed using Lipofectamine reagent (GenePharma Co., Shanghai, China) according to the manufacturer’s instructions. After 48-h transfection, reverse transcriptase (RT)-PCR was used to measure hTERT mRNA expression. The hTERT-2985 sequence was found to reduce the level of hTERT mRNA to the greatest extent and was therefore selected for further study.

### Construction of an hTERT-shRNA eukaryotic expression plasmid

The RNA oligonucleotides 5′-CGGUGUGCACCAACAUCUATT-3′ (sense) and 5′-UAGAUGUUGGUGCACACCGTC-3′ (antisense) were chemically synthesized (GenePharma Co., Shanghai, China) and annealed to form a duplex with a symmetric overhang of two deoxythymidines on the 3′ end (hTERT-siRNA). An siRNA duplex containing the unrelated sequence 5′-GTTCTCCGAACGTGTCACGT-3′ (NC-siRNA) was also synthesized as a negative control for siRNA specificity. To construct a plasmid expressing NC- or hTERT-siRNA for stable transfection, the annealed oligonucleotides 5′-caccgTTCTCCGAACGTGTCACGTcaagagattACGTGACACGTTCGGAGAAtttttt-3′ and 5′-caccgCGGTGTGCACCAACATCTAttcaagagaTAGATGTTGGTGCACACCGttttttg-3′ (target sequences in capital letters) were subcloned into the PGPU6/GFP/Neo vector (GenePharma). This vector expresses coral green fluorescent protein (cGFP) under the control of the CMV promoter to allow monitoring of transfection efficiency. Plasmids expressing NC- and hTERT-siRNA were designated, respectively, PGPU6/GFP/Neo-NC-shRNA (hereafter: NC-shRNA) and PGPU6/GFP/Neo-hTERT-shRNA (hereafter: hTERT-shRNA).

### Transient transfection of SW480 cells

Cultures of 2×10^5^ cells per well were grown in 6-well plates to 90% confluence and transfected with 100 nM NC-shRNA or hTERT-shRNA using Lipofectamine 2000 (Invitrogen, Carlsbad, CA, USA) according to the manufacturer's instructions. In parallel, cells were transfected with the same amount of Lipofectamine 2000 without any shRNA plasmid as a control. Cultures were harvested in triplicate after transfection for 24, 48, and 72 h.

### RT-PCR to measure hTERT mRNA expression after transfection

At each time point indicated above, total RNA was extracted from transfected SW480 cells using Trizol and subjected to RT-PCR as described above.

### Immunocytochemistry to measure hTERT protein expression

SW480 cells were plated on 2.5 cm coverslips at a density of 5×10^5^ cells/well in 6-well plates. After transfection for 24, 48, or 72 h, coverslips were removed and immunostained with the rabbit polyclonal anti-hTERT antibody (1∶300, Santa Cruz Biotechnology, CA, USA) and the Maxvision kit (Maixin, Fuzhou, China). On each coverslip, five views were randomly selected and captured using image analysis software (Leica, Germany). In each view, the grayscale intensity was measured at 10 randomly selected points and averaged. The average grayscale intensity was inversely proportional to the protein level.

### TRAP assay of telomerase activity

Telomerase activity was assayed using a commercial Telomerase PCR ELISA kit (Roche Diagnostics Scandinavia AB, Stockholm, Sweden) according to the manufacturer's instructions. This assay is based on the telomeric repeat amplification protocol [20]. After SW480 cells had been transfected for 48 h with Lipofectamine 2000 alone, NC-shRNA or hTERT-shRNA, or left untransfected in culture medium as a blank control, total cellular proteins were extracted using CHAPS lysis buffer, and a 0.5-µg aliquot was assayed. The assay consisted of a telomerase-primer elongation reaction, followed by 26 PCR cycles. PCR products were detected using an ELISA-based color reaction [20].

Telomerase activity was expressed as an adjusted absorbance, given by the absorbance in arbitrary units at 450 nm minus the absorbance at the reference wavelength of 690 nm. According to the manufacturer's instructions, the maximum adjusted absorbance for the negative control should be 0.25, while the adjusted absorbance for the positive control reaction supplied in the kit should be >1.5 after 20 min of reaction. Samples were defined as telomerase-positive if adjusted absorbance was >0.2.

### Flow cytometry to measure cell growth and proliferation

SW480 cells transfected for 48 h with Lipofectamine 2000 alone, NC-shRNA or hTERT-shRNA, or left untransfected in culture medium as a blank control, were harvested, washed with phosphate-buffered saline (PBS), and fixed overnight with 66% ethanol. Cells were centrifuged and washed with PBS, then incubated with 50 µg/mL propidium iodide and 2.5 µg/mL RNase in PBS for 30 min at room temperature. DNA content was analyzed by flow cytometry at an emission wavelength of 488 nm using Multicycle software (BD Biosciences, USA) [21]. The proliferation index (PI) was calculated according to the formula:

PI  =  [S + (G2/M)]/{(G0/G1) + [S + (G2/M)]} ×100%.

### Flow cytometry to measure apoptosis

Apoptosis was measured by double-staining cells for annexin V and DNA (propidium iodide) and analyzing them by flow cytometry. SW480 cells transfected for 48 h with Lipofectamine 2000 alone, NC-shRNA or hTERT-shRNA were cultured in the presence of the indicated concentrations of pristimerin, harvested, washed with PBS, and incubated in Annexin V Binding Buffer (BD Pharmingen) containing 0.3% FITC-conjugated Annexin V for 15 min at room temperature. The cells were washed and resuspended in Annexin V Binding Buffer. Propidium iodide was added just before flow cytometry [21]–[22]. Data were analyzed using FACSDiva Software (BD Biosciences).

### TUNEL assay to measure apoptosis

The Dead End Colorimetric TUNEL System (Kaiji, Nanjing, China) was used to measure apoptosis in SW480 cultures transfected with Lipofectamine 2000 alone, NC-shRNA or hTERT-shRNA, as well as in nude mice injected with saline, NC-shRNA or hTERT-shRNA plasmids (see below). For in vitro experiments, SW480 cells were plated onto 2.5-cm coverslips and transfected for 48 h, after which coverslips were removed and fixed for the TUNEL assay according to the manufacturer’s protocol. For in vivo experiments, tumor tissue slices (5 µm thick) were prepared. Slides were fixed in 10% PBS-buffered formalin (Kaiji, Nanjing, China) and permeabilized with proteinase K (Kaiji). Then slides were processed as described by the manufacturer.

Slides were assayed for apoptotic cells following the manufacturer’s protocol. Briefly, recombinant terminal deoxynucleotidyl transferase was used to add biotinylated nucleotides to the DNA, sodium chloride and sodium citrate were added to stop the reaction, and then the slides were incubated with horseradish peroxidase-labeled streptavidin. Finally, the slides were incubated with a mixture of hydrogen peroxide, diaminobenzidine chromogen, and peroxidase substrate in order to stain the nuclei dark brown. Stained slides were then analyzed by light microscopy using an image capture system (Leica, Germany). At least 3 high-magnification fields were examined on each slide (>500 cells altogether), and the apoptotic index (AI) was calculated as the percentage of observed cells staining TUNEL-positive.

### Transmission electron microscopy (TEM) to assess apoptotic morphology

SW480 cells were transfected for 48 h with Lipofectamine 2000 alone, NC-shRNA or hTERT-shRNA and then harvested. Cells were pelleted, immediately fixed in 3% (v/v) glutaraldehyde, postfixed in 1% (v/v) osmium tetroxide and stained with 4.8% uranyl acetate. After dehydration through a graded series of alcohol solutions, samples were rinsed in propylene oxide and impregnated with epoxy resin. The ultrathin sections were treated with uranyl acetate and lead citrate to provide contrast for TEM, which was performed using a JEM-1230 microscope (JEOL, Japan).

### Measurement of mitochondrial membrane potential (MMP) to assess apoptosis

MMP was measured using rhodamine 123 fluorescent dye as an indicator (CAS 83702; Sigma, Shanghai, China) as previously described [23]. This dye has an excitation maximum at 488 nm and an emission maximum at 526 nm. In this approach, MMP is assumed to vary inversely with rhodamine 123 fluorescence. SW480 cells were transfected for 48 h with Lipofectamine 2000 alone, NC-shRNA or hTERT-shRNA, then washed twice with PBS to remove the dead cells and incubated with rhodamine 123 (0.1 µg/mL) at 37°C for 30 min. Rhodamine 123 fluorescence intensity was measured using confocal scanning microscopy (NIKON-AE, Japan). For each experimental condition, mean fluorescence intensity was determined for 500 cells.

### hTERT knock-down in a nude mouse tumor model

SW480 cells were subcutaneously injected into the right flank of nude mice at a dose of 5×10^7^ cells per mouse in 200 µl of DMEM diluted 1∶2 in PBS [24]. When the tumor nodules had grown to 7 mm, the mice were randomly divided into three groups of 8 animals, with each group receiving a total of 6 intratumor injections of normal saline, 20 µg NC-shRNA or 20 µg hTERT-shRNA every 2 days. The animals were observed for 6 days after the last shRNA injection [25]. Tumor length (L), width (W) and diameter were measured twice a week; tumor volume (cm^3^) was calculated using the formula W^2^× (L/2) [26]. The mice were killed and neutral formalin-fixed tumor samples were stained with hematoxylin-eosin and examined by light microscopy.

Levels of hTERT mRNA and protein in the three groups of nude mice were determined, respectively, by RT-PCR and immunohistochemistry as described above. Telomerase activity and extent of apoptosis using the TUNEL assay were also measured as described above.

### Statistical analysis

Results were expressed as mean ± SD. All statistical analyses were carried out using SPSS 16.0 ( IBM, USA). Differences between treatment conditions were assessed for statistical significance using one-way ANOVA, followed by the LSD or Dunnett’s t test method. The threshold for significance was defined as *P*<0.05.

## Results

### Endogenous hTERT expression in colon cancer cell lines

**Figure 1:**
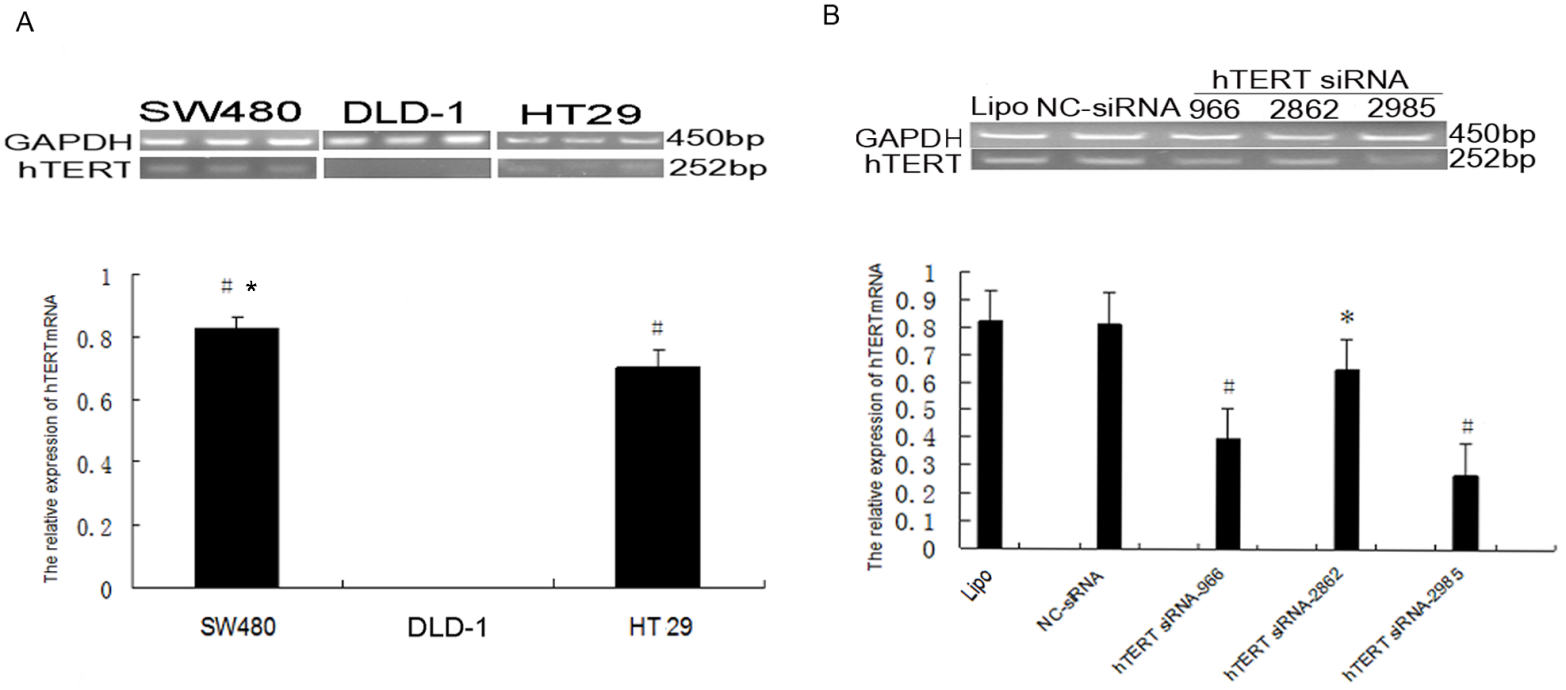
Screening colon cancer cell lines and effective hTERT-siRNA. (**A**) Endogenous hTERT mRNA expression in SW480, DLD-1 and HT29 cell lines. There were significant differences among SW480, HT29 and DLD-1 cells (^#^ *P*<0.01), and between SW480 and HT29 cells (*P<0.05). (**B**) Potency of hTERT siRNAs in SW480 cells. Levels of hTERT mRNA were significantly lower in the cells transfected with hTERT siRNAs than in cells transfected with negative control (NC) shRNA or Lipofectamine alone (Lipo). *P<0.05, ^#^ *P*<0.01, compared with the NC-shRNA and Lipo groups. Results are mean ± SD from three independent experiments.

First we screened the SW480, DLD-1, and HT29 lines to identify which had sufficiently high hTERT mRNA expression to facilitate RNA interference and analysis of the effects on cell growth. RT-PCR showed that hTERT mRNA levels were barely detectable in DLD-1 cells and significantly higher in SW480 cells than in HT29 cells (0.827±0.037 *vs.* 0.705±0.051, *P<*0.05; Fig. 1A). Therefore SW480 cells were chosen for further study.

### Screening for the most potent hTERT siRNA in SW480 cultures

Three hTERT-siRNAs (hTERT-966, hTERT-2862, hTERT-2985) and an unrelated siRNA as a negative control (NC-siRNA) were transiently transfected into SW480 cells to identify the siRNA that most potently suppressed hTERT mRNA level, as well as to confirm the hTERT specificity of our siRNA approach. Control cells transfected with Lipofectamine reagent alone (“Lipo”) or with NC-siRNA showed similar levels of hTERT mRNA (0.823±0.025 and 0.815±0.043, respectively; *P*>0.05). Transfection with any of the three hTERT-siRNAs led to significantly lower hTERT mRNA levels than transfection with NC-siRNA or Lipo alone (Fig. 1B): hTERT-966, 0.395±0.075; hTERT-2862, 0.650±0.064; hTERT-2985, 0.270±0.056 (all *P*<0.05). Of the three hTERT siRNA sequences tested, hTERT-2985 was identified as the most potent and was therefore used to construct an hTERT-shRNA eukaryotic expression plasmid for further in vitro and in vivo studies.

### Expression of hTERT-shRNA in SW480 cells down-regulates mRNA and protein expression

**Figure 2:**
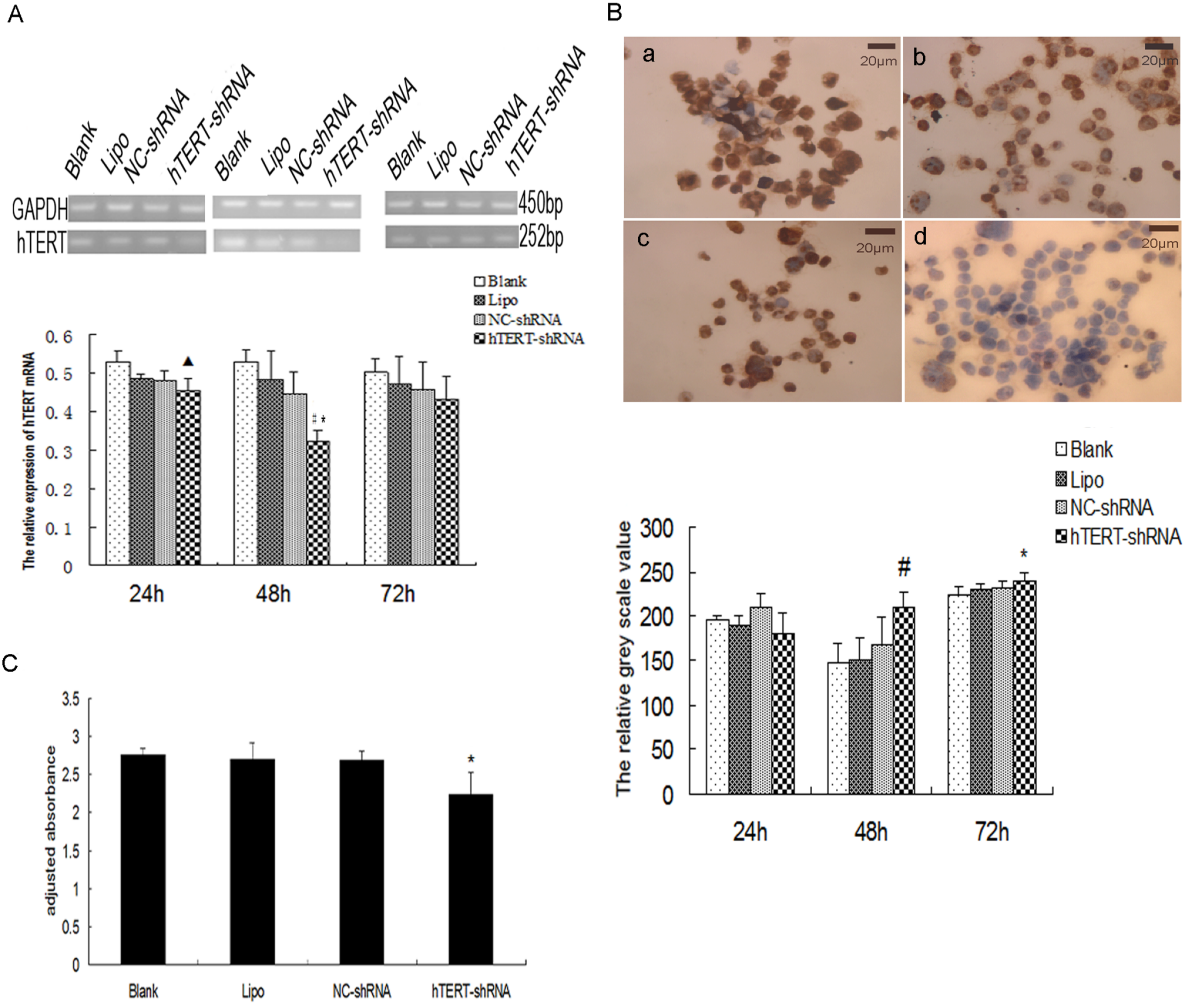
hTERT-shRNA inhibits hTERT expression and telomerase activity *in vitro*. (**A**) RT-PCR analysis of hTERT mRNA expression in SW480 cultures after mock-transfection with culture medium alone (Blank), Lipofectamine alone (Lipo), negative control siRNA (NC-shRNA), or hTERT-shRNA. Levels of mRNA levels were significantly lower after 48-h transfection with hTERT-shRNA than after control transfections. ^▴^ *P*<0.05, ^#^ *P*<0.01, compared with the Blank; *P<0.05, compared with Lipo and NC-shRNA. (**B**) Immunocytochemistry of hTERT protein expression. (a) Blank cells, (b) Lipo cells, (c) NC-shRNA cells, and (d) hTERT-shRNA cells. All views, ×400. Higher greyscale value indicates lower protein level. Protein levels were significantly lower after 48-h transfection with hTERT-shRNA than after control transfections. ^#^ *P*<0.01, compared with the other groups; *P<0.05, compared with the Blank. (**C**) HTERT down-regulation inhibits telomerase activity. Telomerase activity [adjusted absorbance (450 nm–630 nm)] was significantly lower in cells transfected with hTERT-shRNA than in control cells. *P<0.05, compared with the Lipo and Blank controls. Results are mean ± SD from three independent experiments.

We transfected SW480 cells with hTERT-shRNA or NC-shRNA for 24, 48 and 72 h, then measured expression of hTERT mRNA and protein. At 48-h transfection, control cultures showed similar mRNA levels: culture medium alone (Blank), 0.530±0.032; Lipofectamine alone (Lipo), 0.483±0.075; NC-shRNA, 0.447±0.058. Levels of mRNA were significantly lower after 48-h transfection with hTERT-shRNA (0.322±0.030; *P*<0.05 or *P*<0.01; Fig. 2A). In fact, the reduction in hTERT mRNA was already observable after 24-h transfection. However, no differences in hTERT mRNA expression were found among the groups after 72-h transfection (Fig. 2A).

Similar results were obtained when we examined hTERT protein expression. While the three control transfections led to strong yellow nuclear membrane staining with abundant brown particles in the nuclei, transfection with hTERT-shRNA led to much weaker staining (Fig. 2B, a–d). Quantitation of greyscale intensities, which varied inversely with the level of hTERT protein, showed that 48-h transfection with hTERT-shRNA led to much lower protein levels (209.600±17.101) than transfection with culture medium alone (146.500±22.325), Lipofectamine alone (151.800±24.478) or NC-shRNA (167.350±37.754) (*P*<0.01; Fig. 2B). After 72-h transfection, although a significant difference persisted between hTERT-shRNA and the blank control (239.500±9.546 vs. 223.950±10.259, *P*<0.05; Fig. 2B), there was no significant difference between hTERT-shRNA and NC-shRNA. These results likely reflect the fact that our expression vector is intended for short-term expression studies because it does not integrate into the host genome. Therefore we performed all further in vitro experiments out to 48 h.

### hTERT down-regulation inhibits telomerase activity in SW480 cells

Similar levels of telomerase activity were observed in SW480 cells transfected for 48-h with culture medium alone (2.756±0.089), Lipofectamine alone (2.693±0.225) or NC-shRNA (2.691±0.119). In contrast, 48-h transfection with hTERT-shRNA led to significantly lower telomerase activity (2.242±0.284; *P<*0.05; Fig. 2C).

### hTERT down-regulation reduces growth and proliferation of SW480 cells

**Figure 3:**
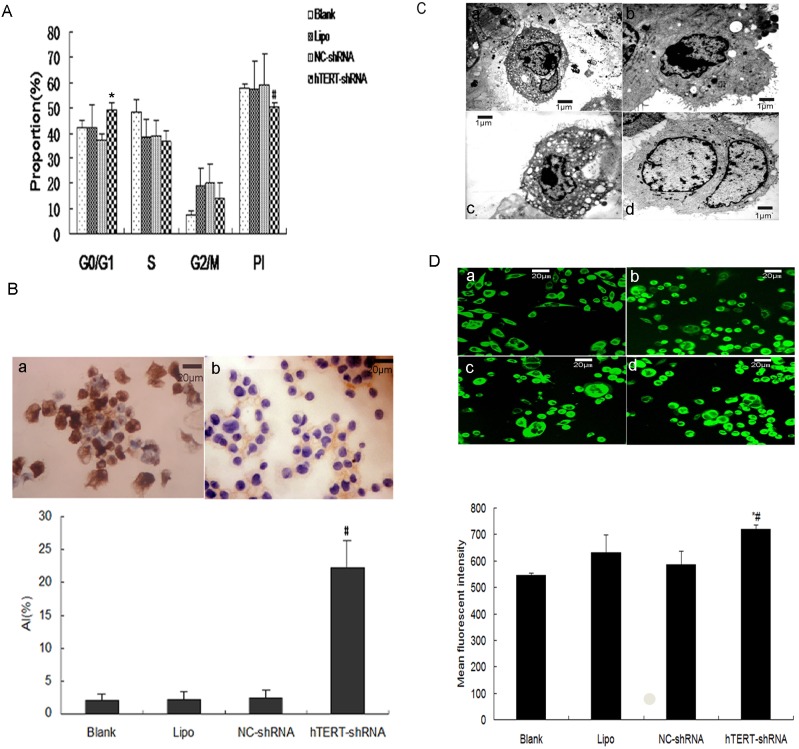
hTERT down-regulation reduces SW480 growth and proliferation and increases apoptosis at 48 h *in vitro*. (**A**) hTERT-shRNA reduces growth and proliferation of SW480 cells. The proportion of cells in G0/G1 phase was significantly higher and the proliferation index was much lower after transfection with hTERT-shRNA than after control transfections. *P<0.05, compared with the other three groups; ^#^ *P*<0.05, compared with NC-shRNA. (**B**) hTERT-shRNA increases apoptosis of SW480 cells. Cells were transfected with (a) hTERT-shRNA or (b) NC-shRNA, stained with diaminobenzidine (brown) and counter-stained with hematoxylin (blue) to label nuclei. Images are representative results from three independent experiments. Magnification, ×400. The apoptotic index (AI) was significantly higher in cells transfected with hTERT-shRNA. ^#^ *P*<0.01, compared with the other three groups. (**C**) Transmission electron microscopy analysis of SW480 cells transfected with (a-c) hTERT-shRNA or (d) NC-shRNA. Apoptotic morphology was visible in cells transfected with hTERT-shRNA. Magnification, ×3800. (**D**) Rhodamine 123 fluorescence intensity analysis of SW480 cells transfected with (a) culture medium alone (Blank), (b) Lipofectamine alone (Lipo), (c) NC-shRNA or (d) hTERT-shRNA. Stained cells were analyzed by confocal scanning microscopy; higher fluorescence intensity indicates lower mitochondrial membrane potential. Magnification, ×400. Cells transfected with hTERT-shRNA showed significantly higher rhodamine 123 fluorescence than did control cells. *P<0.05, compared with NC-shRNA or Lipo; ^#^ *P*<0.01, compared with Blank. Results are the mean ± SD from three independent experiments.

The proportion of cells in G0/G1 phase was significantly higher after 48-h transfection with hTERT-shRNA (49.37±1.63%) than after transfection with culture medium alone (42.27±2.76%), Lipofectamine alone (42.43±4.67%) or NC-shRNA (37.07±1.53%, P<0.05). At the same time, the proliferation index was much lower after transfection with hTERT-shRNA (50.6±1.57%) than after transfection with NC-shRNA (59.03±5.86%, P<0.05); the index was similar for all three control groups (Fig. 3A).

### hTERT down-regulation increases apoptosis of SW480 cells

In contrast to control-transfected cells, cells transfected with hTERT-shRNA were shrunken in appearance; they showed deep-brown staining in the cell membrane, cytoplasm or nucleus; and they occasionally contained apoptotic bodies (Fig. 3B, a–b). TUNEL staining showed that the proportion of apoptotic cells was much higher after transfection with hTERT-shRNA [Apoptosis index (AI)  = 22.2±4.25%] than after transfection with culture medium alone (1.98±1.05%), Lipofectamine alone (2.2±1.20%) or NC-shRNA (2.4±1.25%; all *P*<0.01; Fig. 3B). TEM analysis also revealed that a large proportion of cells transfected with hTERT-shRNA had apoptotic morphology, including reduced volume, reduced numbers of protrusions and microvilli, and uneven chromatin accumulation under the nuclear membrane (Fig. 3C, a). Some cell nuclei were crescent-shaped, circular or chunky, and many cells contained numerous vacuoles (Fig. 3C, b–c). In contrast, control-transfected cells showed normal internal structure (Fig. 3C, d).

As an additional measure of apoptosis, we used rhodamine 123 as an index of MMP (Fig. 3D, a–d); in this assay, higher dye intensity indicates lower MMP, which is associated with apoptosis. Cells transfected with hTERT-shRNA showed significantly higher rhodamine 123 fluorescence (719.998±17.083) than did cells transfected with culture medium alone (545.833±6.811, *P*<0.01), NC-shRNA (585.361±51.781, *P*<0.05) or Lipofectamine alone (632.169±65.635, *P*<0.05) (Fig. 3D).

### hTERT-shRNA inhibits tumor cell growth *in vivo*

**Figure 4:**
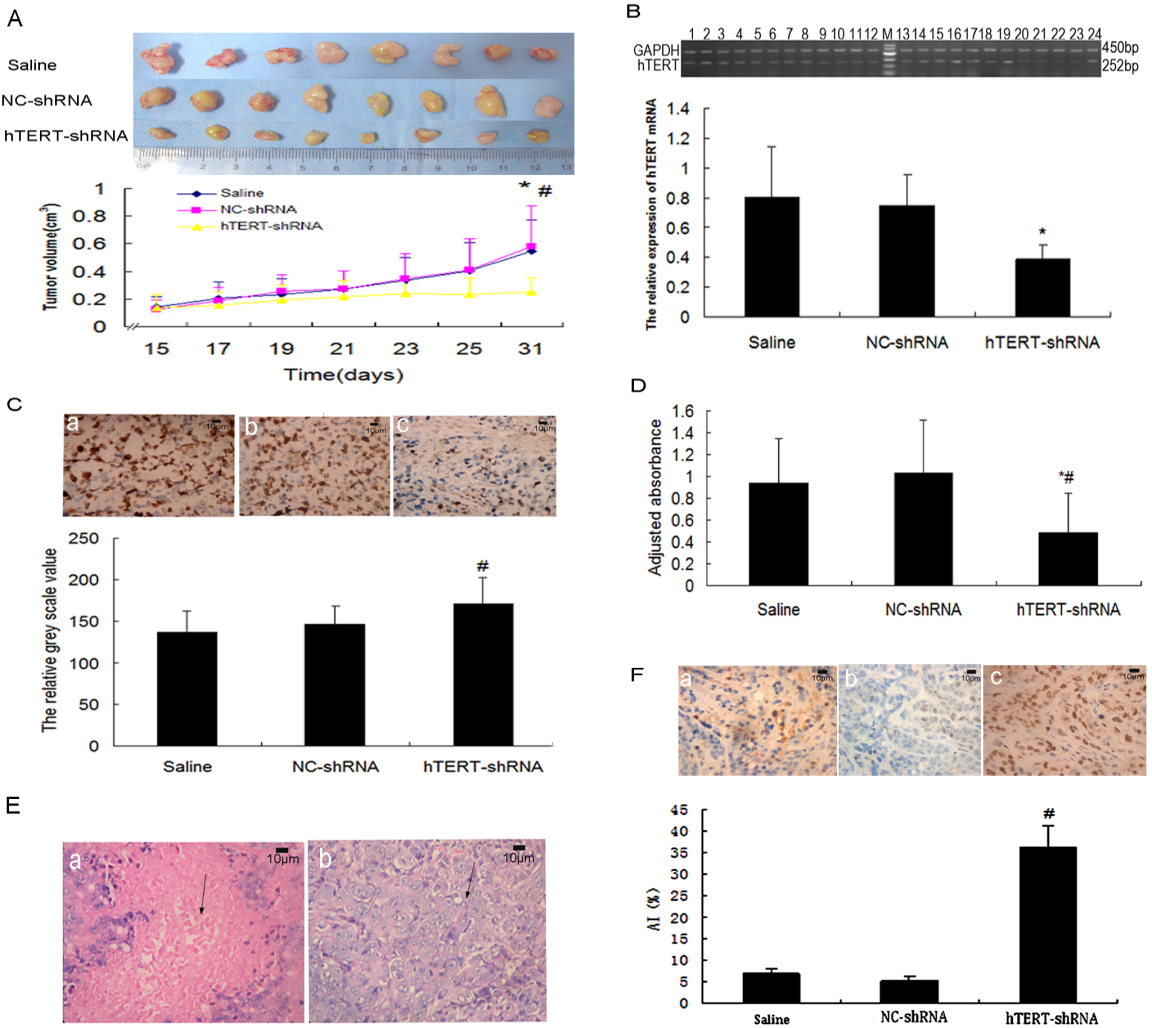
Effects of hTERT-shRNA on SW480 tumor growth, apoptosis and telomerase activity *in vivo*. (**A**) hTERT-shRNA inhibits tumor cell growth. Tumor growth at 31 days was significantly slower in tumors treated with hTERT-shRNA than in tumors treated with saline (*P<0.05) or NC-shRNA (^#^ *P*<0.01). (**B**) Histopathological examination of SW480 tumor tissue grafted onto nude mice and treated with hTERT-shRNA. Tumor tissue was biopsied at 31 days and stained with hematoxylin-eosin. The arrow in (a) indicates sheet necrosis; the arrow in (b) indicates inversion of the nucleoplasm to cytoplasm volume, together with mitotic pathology. Magnification, ×200. (**C**) RT-PCR analysis to measure hTERT mRNA expression in tumor tissue treated with saline (lanes 1–8), NC-shRNA (lanes 9–16), or hTERT-shRNA (lanes 17–24). M, 100-bp DNA ladder. The relative expression of hTERT mRNA was significantly lower in the hTERT-shRNA mice. *P<0.05, compared with the saline and NC-shRNA groups. (**D**) Immunocytochemistry of hTERT protein (stained brown) in tumor tissue treated with (a) saline, (b) NC-shRNA, or (c) hTERT-shRNA. Magnification, ×200. Higher greyscale values indicate lower protein levels. The relative expression of hTERT protein was significantly lower in hTERT-shRNA mice. #P<0.01, compared with the saline and NC-shRNA groups. (**E**) Effect of hTERT-shRNA on telomerase activity in vivo. The adjusted absorbance (450 nm - 630 nm) was significantly lower in tumors treated with hTERT-shRNA than in tumors treated with saline (*P<0.05) or NC-shRNA (^#^ *P*<0.01). (**F**) Effects of hTERT-shRNA on apoptosis. Tumors were injected regularly with (a) saline, (b) NC-shRNA or (c) hTERT-shRNA, then biopsied and stained with diaminobenzidine (brown); nuclei were counter-stained with hematoxylin (blue). Images are representative results of three independent experiments. Magnification, ×200. The apoptotic index (AI) was significantly higher in tumors treated with hTERT-shRNA than in tumors treated with saline or NC-shRNA. ^#^ *P*<0.01, compared to the two control groups.

A xenograft tumor model was established by injecting nude mice with SW480 cells and then injecting the resulting tumors with NC-shRNA or hTERT-shRNA. Periodic measurement of tumor size showed that at 31 days, tumors treated with hTERT-shRNA (0.248±0.102 cm^3^) were significantly smaller than tumors treated with saline (0.543±0.230 cm^3^, *P*<0.05) or NC-shRNA (0.580±0.293 cm^3^, *P*<0.01; Fig. 4A).

### hTERT-shRNA inhibits hTERT expression and telomerase activity *in vivo*

After 31 days of treatment with saline, NC-shRNA, or hTERT-shRNA, relative expression of hTERT mRNA was significantly lower in the hTERT-shRNA mice (0.388±0.093) than in the saline (0.809±0.335) or NC-shRNA animals (0.750±0.206; *P*<0.05; Fig. 4B). Similarly, cells positive for dark brown-yellow hTERT protein staining in the cytoplasm and nucleus were more abundant in saline and NC-shRNA mice (Fig. 4C, a-b) than in the hTERT-shRNA animals (Fig. 4C, c). Similar results were obtained when comparing expression of hTERT protein: relative expression was significantly lower in hTERT-shRNA mice (171.42±30.94) than in saline (137.35±25.49) or NC-shRNA animals (146.89±21.43) (*P*<0.01; Fig. 4C). The two control groups were similar in their levels of hTERT mRNA and protein (*P*>0.05).

Consistent with our measurements of hTERT mRNA and protein, we found telomerase activity to be significantly lower in the hTERT-shRNA mice (0.484±0.359) than in mice treated with saline (0.940±0.404, *P*<0.05) or NC-shRNA (1.030±0.483, *P*<0.01; Fig. 4D).

### hTERT-shRNA promotes tumor cell apoptosis *in vivo*

After hematoxylin-eosin staining, tumor tissue from nude mice injected with hTERT-shRNA showed sheet necrosis, disappearance of intracellular sructures, and cell sputtering (Fig. 4E, a). In contrast, tumor tissue from control nude mice injected with saline or NC-shRNA showed the expected large and irregular cell shapes, inversion in the ratio of nucleoplasm to cytoplasm volume, and mitotic pathologies (Fig. 4E, b). These findings were supported by TUNEL staining, which provided semi-quantitative information about apoptosis levels. In the TUNEL assay, apoptotic cells are yellowish-brown and they show a condensed nucleus and cytoplasm as well as irregular cell shape; healthy cells, in contrast, show normal morphology and weak or no brown staining. TUNEL-positive cells were more abundant in tumor tissue from hTERT-shRNA mice than in tissue from the two control groups (Fig. 4F, a-c). Indeed the apoptosis index (AI) was significantly higher in tumor tissue from hTERT-shRNA mice (36.3±5.05%) than in tumor tissue of saline mice (6.95±1.07%) or NC-shRNA mice (5.25±1.06%, all *P*<0.01; Fig. 4F).

## Discussion

The advent of RNAi-directed gene knock-down techniques has sparked a revolution in somatic cell genetics, allowing the inexpensive and rapid analysis of gene function in mammals, as well as screening for gene therapy targets to treat disease. In fact, RNAi has been shown in several studies to be more effective and longer-lasting than antisense approaches in both cell culture and nude mice [27]–[28]. Here we screened hTERT siRNA sequences to identify one that effectively and specifically down-regulates expression of hTERT mRNA and protein. We showed that expressing the optimal sequence in SW480 cultures and SW480-derived tumors in nude mice inhibits telomerase activity, promotes tumor apoptosis and inhibits tumor growth. Here we provide what is, to our knowledge, the first qualitative and quantitative evidence for a role of hTERT in apoptosis of colon cancer cells, the first report of telomerase-targeting gene therapy in SW480 cells, and perhaps the most complete assessment so far of an RNA interference-based reagent against colorectal cancer. These findings confirm the potential of gene therapy targeting hTERT to treat human colorectal cancer.

Our findings confirm and extend similar work in the HCT116 human colorectal cancer cell line [29], in which the authors used a different sequence than ours to knock-down hTERT expression by RNAi; this inhibited telomerase activity and cell growth in cultures and in HCT116 tumors in nude mice. We wished to study the cellular effects of hTERT inhibition in SW480 cells in even more detail, so we examined apoptosis levels in cultures by electron microscopy, TUNEL assay and MMP measurement, as well as in SW480 tumors by TUNEL assay. Our results in SW480 cells, together with those in HCT116 cells [29], indicate that colon cancer should be added to the list of malignancies that can be inhibited by RNAi targeting hTERT in vitro and in vivo; this list already includes lung cancer, oral squamous cell carcinoma, erythroleukemia, hepatocellular carcinoma, cervical epithelial squamous cancer, and gastric cancer [30]–[34], [29]. Our SW480 model system may prove particularly useful for further therapeutic research because the cells are derived from advanced (grade 3–4) colon adenocarcinoma, they are fairly homogeneous (mostly epithelial cells), they produce carcinoembryonic antigen (CEA), and they express hTERT mRNA at high levels. Thus these cells present several advantages over other colon cancer cell lines like HT29 or DLD-1.

Since regulation of telomerase activity is a complex process involving transcriptional control, post-translational modification, positively and negatively acting factors and alternative splicing [9], [35], we wanted to ensure that our knock-down of hTERT mRNA and protein translated to lower telomerase activity. Therefore we measured the enzyme activity directly using the TRAP method [20]. As expected, we found that hTERT-shRNA significantly decreased telomerase activity, showing a direct link between enzyme inhibition and tumor cell growth inhibition. Whether this link involves changes in telomere length is unclear. Previous studies in the HCT116 colon cancer cell line [29] and in other cell types [36]–[37] have shown that down-regulation of telomerase activity leads to tumor growth inhibition independent of changes in telomere length. Consistent with a possible telomere length-independent mechanism, we found that hTERT knock-down induced G0/G1 arrest in SW480 cells, which may reflect the reported ability of hTERT to associate with human telomeres and thereby enhance genomic stability and stimulate DNA repair [38]. Future studies should probe directly whether RNA interference against hTERT affects telomere length.

In addition to acting directly on cell cycle progression, hTERT knock-down induced apoptosis in SW480 cells in vitro and in vivo, based on microscopic examination of morphology, TUNEL staining, and MMP measurements. Structural and functional changes in mitochondria [39]–[40], together with a decrease in MMP [41], occur early in apoptosis, and we observed swollen mitochondria and significantly reduced MMP in tumor cells treated with hTERT-shRNA, but not in tumor cells treated with an unrelated shRNA. The pro-apoptotic effects of hTERT knock-down may be mediated by metabolic changes, as observed in the apoptotic processes of diverse normal and diseased cells, including tumors [42]–[43]. These findings that RNA interference against hTERT inhibits the growth of SW480 cells by affecting apoptosis provide qualitative and quantitative evidence of a previously unrecognized role for hTERT in apoptosis of colon cancer cells.

RNAi against hTERT appears to work by different mechanisms in different cell types. Whereas we found hTERT inhibition to increase apoptosis and block cells in G0/G1 phase, similar RNAi against hTERT increased telomere attrition in HT29 cells [44], increased early but not late apoptosis in SiHA cells and blocked them in S phase [34], and inhibited migration and invasion of HCT116 cells [29]. RNAi against hTERT in K562 cells led to an increase in apoptosis only when the cultures were serum-deprived [32]. Appreciating the diversity of these mechanistic pathways will be important for future work aimed at understanding the role of hTERT in tumor growth and its usefulness as a gene therapy target.

In our SW480 system, we examined only the effects of exposing cell cultures directly to siRNA or of injecting tumors with siRNA. Therefore future work should determine how best to deliver the siRNAs to tumor cells in the clinic. Formulating the siRNA with bioreducible cationic polymers, for example, shows substantial promise [45], [33], as does packaging in adenovirus [31]. Combining siRNA against hTERT with other therapies, such as radiation treatment [46], may also be appropriate. In this way, the present study opens the door wider for applied mechanistic and pharmacologic studies of hTERT-based RNAi in colon cancer.

## Conclusions

We have designed a novel hTERT shRNA and demonstrated its effectiveness, in vitro and in vivo, for inhibiting hTERT expression, telomerase activity, and tumor cell growth. Using this system, we provide evidence for a role of hTERT in apoptosis and in colorectal cancer progression. Mechanistic studies should examine whether hTERT down-regulation inhibits growth by a telomere-dependent or -independent mechanism, and applied studies should determine the optimal dosing and delivery methods for safe and effective tumor growth inhibition. Our results, together with previous work, suggest that gene therapy targeting hTERT may serve as an alternative or complement to surgery for treating colorectal cancer.
